# MRI findings in Hirayama disease

**DOI:** 10.4103/0971-3026.73528

**Published:** 2010-11

**Authors:** Monali Raval, Rima Kumari, Aldrin Anthony Dung Dung, Bhuvnesh Guglani, Nitij Gupta, Rohit Gupta

**Affiliations:** Department of Neuroradiology, Institute of Human Behaviour and Allied Sciences (IHBAS), Delhi, India; 1 Department of Neurology, Institute of Human Behaviour and Allied Sciences (IHBAS), Delhi, India; 2 Department of Neuroradiology, Focus Imaging and Research Institute, IHBAS Campus, Delhi, India; 3 Department of Neuroradiology, B.K. Medical Centre, Delhi, India

**Keywords:** Flexion MR, Hirayama disease, 3D-FIESTA sequence

## Abstract

The objective of the study was to study the magnetic resonance imaging (MRI) features of Hirayama disease on a 3 Tesla MRI scanner. Nine patients with clinically suspected Hirayama disease were evaluated with neutral position, flexion, contrast-enhanced MRI and fast imaging employing steady-state acquisition (FIESTA) sequences. The spectrum of MRI features was evaluated and correlated with the clinical and electromyography findings. MRI findings of localized lower cervical cord atrophy (C5-C7), abnormal curvature, asymmetric cord flattening, loss of attachment of the dorsal dural sac and subjacent laminae in the neutral position, anterior displacement of the dorsal dura on flexion and a prominent epidural space were revealed in all patients on conventional MRI as well as with the dynamic 3D-FIESTA sequence. Intramedullary hyperintensity was seen in four patients on conventional MRI and on the 3D-FIESTA sequence. Flow voids were seen in four patients on conventional MRI sequences and in all patients with the 3D-FIESTA sequence. Contrast enhancement of the epidural component was noted in all the five patients with thoracic extensions. The time taken for conventional and contrast-enhanced MRI was about 30–40 min, while that for the 3D-FIESTA sequence was 6 min. Neutral and flexion position MRI and the 3D-FIESTA sequence compliment each other in displaying the spectrum of findings in Hirayama disease. A flexion study should form an essential part of the screening protocol in patients with suspected Hirayama disease. Newer sequences such as the 3D-FIESTA may help in reducing imaging time and obviating the need for contrast.

## Introduction

Non progressive juvenile spinal muscular atrophy, also known as Hirayama disease, is characterized by the insidious onset of unilateral or asymmetric oblique amyotrophy that affects the C7, C8 and T1 myotomes. It occurs in young men and is characteristically associated with cold paresis. The onset is insidious, and is characterized by muscle weakness and atrophy in the hand and forearm, with sparing of the brachioradialis, giving the characteristic appearance of oblique amyotrophy.[1] The amyotrophy is unilateral in most patients, asymmetrically bilateral in some and rarely symmetric. Hirayama disease is a benign, nonprogressive motor neuron disease. Chronic microcirculatory changes in the territory of the anterior spinal artery induced by repeated or sustained flexion account for the necrosis of the anterior horns of the lower cervical cord, which is the hallmark on pathology.[1,2] A spectrum of diagnostic magnetic resonance imaging (MRI) features has been described in the literature. The present study reviews the MRI features in Hirayama disease in the neutral and flexion positions and the use of the newer 3D fast imaging employing steady-state acquisition (FIESTA) sequence in a 3 Tesla MRI scanner.

## Materials and Methods

The study was conducted in a tertiary-care hospital in India. Nine patients with a clinical suspicion of Hirayama disease were evaluated.

The basis of the diagnosis was as follows: (1) chronic weakness and atrophy of the distal upper limb(s), (2) insidious onset in the teen years or in the early 20s, (3) irregular coarse tremors in the fingers of the affected hand(s), (4) absence of substantial sensory deficits, reflex abnormalities, as well as of cranial nerve, pyramidal tract lower limb, sphincter or cerebellar involvement, (5) non progressive course and arrest of disease within a few years of onset and (6) electromyography-based evidence of chronic denervation in the clinically or subclinically affected muscles and absence of objective sensory loss.

Neurophysiologic workup included nerve conduction study (NCS) and electromyography (EMG).

The MRI imaging was performed on a 3 Tesla Signa HDX system (GE, Milwaukee, WI, USA). The neutral position MRI protocol included transverse and sagittal T1W sequences (fast-spin echo, repetition time in ms/echo time of 400–700/13–20), a transverse T2*W sequence (gradient echo, 2D MERGE with a bandwidth of 31) and a sagittal T2W sequence (fast recovery fast spin echo FRFSE, 2000–2500/90). The flexion imaging protocol consisted of a sagittal T1W MRI sequence (400–700/13–20), a transverse T2W sequence (FRFSE, 2000–2500/90) and a transverse T2*W sequence (gradient echo, 2D MERGE with a bandwidth of 31), with maximum flexion obtained using a positioning sponge. Post gadolinium, transverse and sagittal T1W images were obtained in flexion in all cases. The section thickness was 4 mm, with a 1-mm gap in the sagittal and transverse sequences. In addition to these, a sagittal 3D FIESTA sequence was obtained in the neutral and flexion positions with a repetition time in ms/echo time of 4.5/1.7. Axial reconstructions were obtained and the findings were compared with those obtained using conventional imaging and contrast-enhanced studies. The time taken for the neutral and flexion 3D FIESTA acquisitions was 6 min.

## Results

### Clinical features

Nine patients fulfilled the clinical criteria. All patients were male, with a mean age of onset of 18 years (range, 15–23 years). All patients presented with an insidious onset. Cold paresis was noted in five patients. Muscle weakness and wasting were noted in the right upper limb in four patients and in the left upper limb in four patients, and were asymmetrically bilateral in one patient. Five patients had fine tremulous movements on contractions, five had fasciculations on contraction while one had hyperesthesia on the dorsum of the hand. None had Horner syndrome. The biceps, triceps and supinator reflexes were present in all. The plantar response was flexor and the sensations were normal in all the patients [Table 1].

**Table 1:** Clinical findings

Clinical findings	No. of patients
Insidious onset	9
Cold paresis	5
Oblique amyotrophy	6
Unilateral	8
Fine tremulous movements on contraction	5
Fasciculations on contraction	5
Hyperesthesia in dorsum of hand	1

### Electrophysiological examination

EMG revealed fibrillations in five patients and fasciculations in seven patients with neurogenic changes in the C7, C8 and T1 myotomes in all patients. The motor nerve conduction velocities of the median and ulnar nerves were normal. The compound muscle action potential (CMAP) in the median nerve was reduced in six patients and the sensory nerve action potential (SNAP) in the median nerve was reduced in two patients [Table 2].

**Table 2:** Electrophysiological examination

EMG	
Neurogenic pattern	9
Fibrillation	5
Fasciculation	7
Nerve conduction velocities in median and ulnar nerves	Normal in all patients
CMAP in median nerve	Decreased in six patients
Sensory nerve action potential in median nerve	Reduced in two patients

### Imaging

**Figure 1 (A, B):**
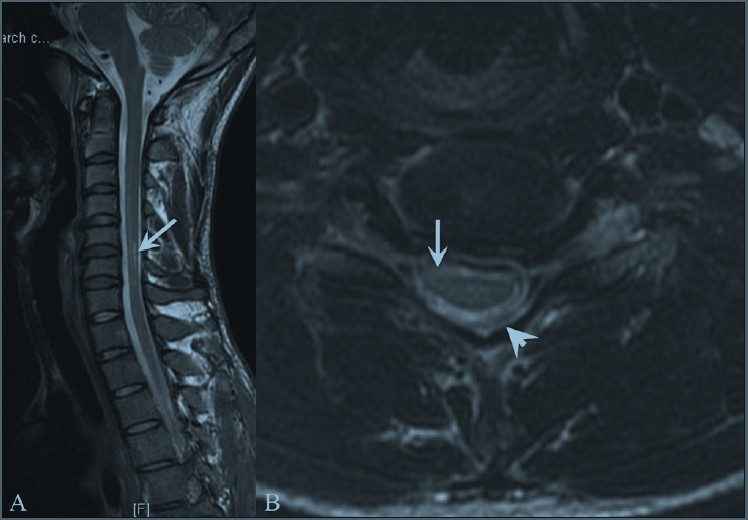
Neutral position T2W sagittal (A) and T2W axial (B) magnetic resonance images show localized cervical cord atrophy at the C5-C7 levels (arrows), which is asymmetrical on the axial image (arrow) with a pear-shaped cross-sectional appearance, with straightening of the cervical curvature and loss of attachment of the dorsal dura (arrowhead)

MRI of the spine revealed localized cord atrophy (C5-C7) and a kyphotic cervical curvature in all patients [Figure 1A]. There was abnormal cord flattening, with a pear-shaped cord on transverse sections affecting the right side in four patients, the left side in four patients and bilaterally asymmetrical in one patient, right more than left. Loss of attachment of the posterior dural sac and subjacent lamina was noted in all patients on neutral position imaging. An intramedullary signal abnormality was seen in four patients [Figure 1B].

**Figure 2:**
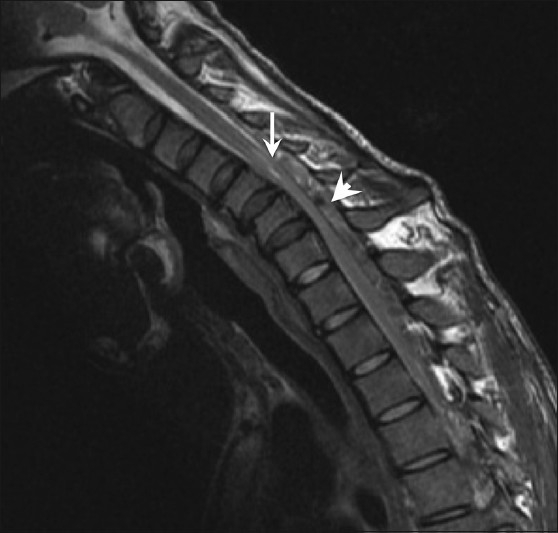
T2W sagittal magnetic resonance image in flexion shows anterior displacement of the dorsal dura (arrow) compressing the thecal sac, with a prominent dorsal epidural compartment (arrowhead)

Flexion imaging showed anterior displacement of the dorsal dura in all patients [Figure 2].

**Figure 3 (A, B):**
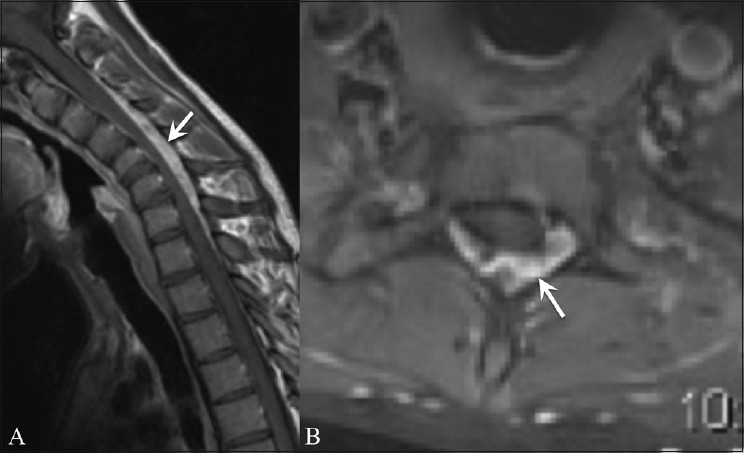
Sagittal flexion (A) and axial (B) contrast-enhanced magnetic resonance images show an enhancing epidural crescent (arrows)

A crescent-shaped epidural mass isointense to the cord on T1WI and hyperintense on T2W images was noted along the posterior aspect of the lower cervical canal (C5-C7) in all patients. Strong homogeneous enhancement was noted on the contrast-enhanced images [Figure 3]. In addition, a thoracic extension of the epidural component was noted in four patients.

**Figure 4 (A, B):**
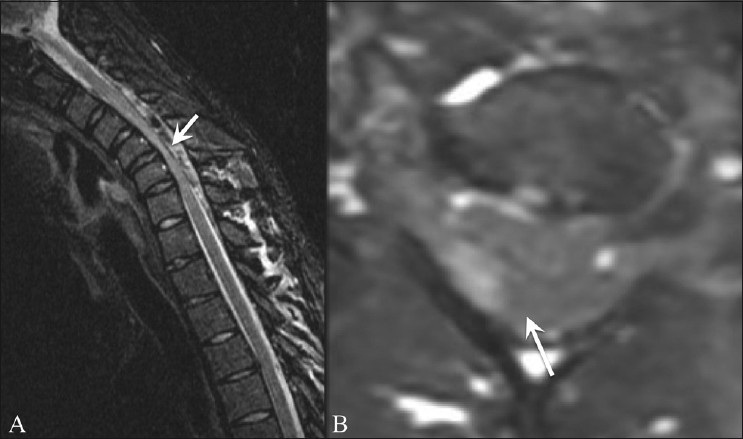
Sagittal flexion (A) and axial (B) 3D fast imaging employing steady-state acquisition magnetic resonance images show prominent flow voids within the dorsal epidural space (arrows) with asymmetric cord compression

Neutral position and flexion 3D-FIESTA images in the sagittal plane with axial reconstructions showed prominent flow voids within the hyperintense, crescentic epidural mass in all patients, thereby reconfirming the findings of both conventional and contrast-enhanced MRI [Figure 4 and Table 3].

**Table 3:** MRI findings

MRI findings	Conventional and contrast MRI	Dynamic FIESTA
Localised cord atrophy	9	9
Abnormal cervical curvature	9	9
Cord Flattening	9	9
Loss of attachment	9	9
Anterior displacement of dorsal dura on flexion	9	9
Enhancing epidural component	9	-
Thoracic extension of epidural component	5	-
Intramedullary signal abnormality	4	4
Prominent flow voids	4	9
Time taken	30-40 minutes	6 minutes

## Discussion

First described by Hirayama *et al*. in 1959, sporadic juvenile muscular atrophy of the upper limb affects young men predominantly, and is characterized by an insidious unilateral or asymmetric bilateral muscular atrophy and weakness of the hand and forearm without sensory or pyramidal signs.[1]

Although the pathomechanism of the disease is debatable, Kikuchi *et al*. believe that an imbalanced growth causes disproportion in the lengths of the vertebral column and the spinal canal contents, resulting in a “tight dural sac.” In the normal spine, the spinal dura is attached at two places – one at the foramen magnum, C2 and C3, and the other at the coccyx – and is anchored to the vertebral canal at the nerve root exits. In healthy subjects, this dura is slack and loosely suspended and consists of several transverse folds, which compensate for the increased length of the cervical canal in flexion. As against this, in Hirayama disease, a short dura cannot compensate for the increased length in flexion and so is displaced anteriorly, with resultant compression of the spinal cord.[3] Toma and Shiozawa proposed that the disproportionate shortening of the dural sac is accentuated during the juvenile growth spurt.[4]

The compression of the lower cervical cord by the posterior dural sac during chronic repeated flexion results in microcirculatory changes in the territory of the anterior spinal artery at the site of the most kyphotic level. Anterior horn susceptibility to ischemia accounts for the atrophy that follows, whereas the white matter is resistant.[2]

Asymmetric cord flattening suggests another predisposing factor – “the posterior epidural ligament factor” – as put forth by Shinomiya *et al*. According to them, two kinds of ligaments between the posterior dura and the ligamentum flavum – one, fine ligaments; and the other, larger ligaments – contribute to resistance against separation of the posterior dura from the ligamentum flavum. Abnormal unequal distribution of the ligaments may be a cause of asymmetric cord compression.[5]

Several conditions like syringomyelia, amyotrophic lateral sclerosis, cervical spondylosis associated with myelopathy, spinal cord tumor and traumatic myelopathy may cause localized amyotrophy of the distal arm, and should be differentiated from Hirayama disease by imaging modalities.[4]

Conventional radiographic studies of the cervical spine are noncontributory and show only loss of cervical lordosis, straight alignment or scoliosis. Mild cord atrophy is noted on lateral myelograms, with forward movement of the posterior dural wall, reduction in the antero-posterior diameter of the dural sac and appearance of a lucent space behind the dural sac on flexion. However, myelography is cumbersome to perform as it is difficult to retain the contrast medium in the cervical subarachnoid space. Computed tomography myelography reveals asymmetrical cord flattening, with the epidural space seen as an area of low density behind the dural sac.[1,6]

As against these modalities, MRI is easy to perform and reveals various findings on neutral and flexion positioning. Localized lower cervical cord atrophy, asymmetric cord flattening, parenchymal changes in the lower cervical cord, abnormal cervical curvature, loss of attachment between the posterior dural sac and subjacent lamina have been described.[7] Among these, localized lower cervical cord atrophy, asymmetric cord flattening and loss of attachment have an accuracy of 80% in identification of the disease; loss of attachment is the most valuable finding for diagnosing Hirayama disease in the neutral position.[7,8]

On flexion MRI, forward migration of the wall of the dura mater is observed with an enlarged posterior epidural space.[1,9,10] A hyperintense, crescentic epidural mass showing curvilinear flow voids and uniform enhancement after administration of contrast is seen in the posterior epidural space.[10] Disappearance of this mass when the neck is in the neutral position suggests congestion of the posterior internal vertebral venous plexus.[3] A combination of three pathophysiological factors is responsible for this meningorachidian venous plexus engorgement. First, the anterior shift of the dural canal is responsible for negative pressure in the posterior spinal canal, with resultant increased flow to the posterior internal vertebral venous plexus.[8] Secondly, the anterior shift of the dura compresses the anterior internal vertebral venous plexus, with resultant increased burden on the posterior internal venous vertebral venous plexus, which leads to its subsequent dilatation.[10] Finally, the venous drainage of the jugular veins is reduced in neck flexion, which in turn impedes the venous return of the internal venous plexus.[8] A dynamic post contrast study as an important method for evaluation of suspected cases of Hirayama disease was stressed by Sonwalkar *et al*.[11] To ensure that this diagnosis is not missed, in patients presenting with focal wasting, after excluding motor neuron disease (MND), if the MRI otherwise looks normal, it should be repeated in flexion as well.

The present study also evaluates the usefulness of the new 3D-FIESTA sequence in the diagnosis of this condition. First described in 1986 as fast imaging employing steady-state precession (FISP), steady-state-free precession (SSFP) sequences are currently known by various synonyms: true FISP, balanced fast-field echo (BFFE) and FIESTA.[12–15] In steady-state imaging, when each subsequent radiofrequency pulse contributes to both longitudinal and transverse magnetization, a steady state is reached. Contrast on these images depends on T2/T1, which is high for cerebrospinal fluid (CSF) and lower for brain, thereby resulting in enhanced CSF intensity as well as an increased CSF-to-tissue contrast.[16]

In our study, flow voids within the epidural mass were poorly visualized on conventional MRI, and its vascular nature could be confirmed only after administration of contrast. Flexion FIESTA in these patients revealed prominent flow voids within the hyperintense crescentic epidural mass in all patients, including within the thoracic extensions in four patients. According to us, the use of the FIESTA study in flexion may obviate the need for a contrast-enhanced study. Being a 3D acquisition, the axial reconstructions can help with the other MRI findings, thereby saving time.

Application of a cervical collar is believed to prevent progression of the disease in early stages, while duroplasty, anterior cervical decompression and reconstructions with tendon transfers have yielded encouraging results in selected patients.[17,18]

Recent studies with magnetization transfer and diffusion tensor imaging (DTI) speculate that mild injury to the corticospinal tracts remains clinically silent due to cortical reorganization, and a correlation between the severity of cord damage and the extent of cortical function changes suggests that the functional changes might have an adaptive role in limiting the clinical consequences of structural cord damage.[19]

To conclude, we suggest the use of neutral and dynamic flexion MRI for the diagnosis of Hirayama disease. The flexion study aids diagnosis in patients of focal wasting if routine imaging is normal. Newer sequences such as 3D FIESTA could be further evaluated as an essential part of the screening protocol in patients with suspected Hirayama disease, thereby obviating the need for contrast administration and saving imaging time.
